# Towards a Sustainable Future: Embedding Planetary Health in Allied Health Professional Education Through the Lens of Indigenous Knowledges

**DOI:** 10.1002/hpja.70195

**Published:** 2026-05-04

**Authors:** Kerstin McPherson, Sarah Barradell, Katrina Li, Michael Watkins, Anna Phillips

**Affiliations:** ^1^ University of NSW, Bidjigal Country Sydney Australia; ^2^ Swinburne University of Technology, Wurundjeri Country Melbourne Australia; ^3^ La Trobe University, Wurundjeri Country Melbourne Australia; ^4^ Adelaide University, Kaurna Country Adelaide Australia

**Keywords:** allied health, Australian aboriginal and Torres Strait islander peoples, curriculum, health occupations, health workforce, planetary health, public health

## Abstract

Planetary health is increasingly recognised as an essential aspect of health professional education, yet its integration within allied health curricula in Australia remains fragmented. This commentary argues that meaningful and sustainable embedding of planetary health requires explicit engagement with Indigenous knowledges. Expanding curricula beyond Western views to include Indigenous eco‐centric worldviews enables a deeper understanding of the interconnections between environmental health, human wellbeing and spiritual identity. We reflect on the influential role of accreditation standards in shaping educational priorities, highlighting the leadership demonstrated by medical and nursing professions and the relative absence of coordinated action across allied health in the context of planetary health. We propose the Aboriginal and Torres Strait Islander Health Curriculum Framework as a culturally grounded lens through which planetary health education can be advanced within allied health education. Aligning planetary health capabilities with the Framework’s five interconnected cultural capabilities offers a practical and transformative pathway to develop culturally capable graduates, strengthen environmental stewardship and promote climate justice. Urgent and inclusive educational reform is required to prepare the future allied health workforce for escalating planetary health challenges.

## 1 Introduction

The health of individuals and communities and the environment are intrinsically linked, making planetary health a critical focus within public health [1]. Some health professions such as medicine and nursing have led the way in thinking about planetary health curricula, setting a precedent for other disciplines. The integration of Indigenous knowledge systems is central to the educational transformation that is needed to understand the interconnectedness of human and environmental well‐being. We argue that the Aboriginal and Torres Strait Islander Health Curriculum Framework [2] might offer all professions a culturally and contextually grounded lens to guide planetary health curricula, help health professions view this as an educational priority, and foster a more inclusive and environmentally responsible health workforce for the future. We offer this argument as an authorship group of health professionals and educators comprising four non‐Indigenous females and one Aboriginal male. We acknowledge our individual and shared positionalities in relation to this topic. We are committed to ongoing critical self‐reflection of our positioning and roles within decolonial health education, striving to privilege Aboriginal narratives. Our approach to this commentary centres on Aboriginal knowledges and voices to contribute to the development of culturally capable health graduates. We also recognise that our diverse backgrounds shape how we engage with and interpret Indigenous knowledge in the context of planetary health.

The increasingly frequent extreme weather events impacting our land and health make this an increasingly important public health agenda, one that requires inter‐professional and inter‐disciplinary collaboration to solve these wicked problems. To respond effectively to both current and future health needs, healthcare practitioners must develop planetary health capabilities, such as systems thinking, not only to provide care but also to contribute to prevention, given that health systems themselves are significant contributors to the climate crisis [3]. Furthermore, equity and environmental justice extend our understanding of health and well‐being by foregrounding the Anthropocene, a term that denotes the scale of human impact on the planet and underscores the inseparability of individual and planetary health [4, 5].

The United Nations through the 2030 Agenda for Sustainable Development [1] and World Health Organisation [6] provides clear guidance for the collaborative inclusion of socio‐ecological determinants of health broadly, with a particular focus on sustainability and addressing new and increasing risks including climate change. In response, many countries and consortiums have developed their own consensus statements and frameworks to provide more specific guidance for universities and healthcare educators. Much of this effort is located in the northern hemisphere, especially the United Kingdom and North America [7, 8]. However, in Australia, and despite national recognition of the connection between health and climate change [9], the response within health professional education appears to be more fragmented, with medicine and nursing leading the way in addressing current and emerging public health challenges.

### 1.1 The Powerful Influence of Accreditation

The medical and nursing professional bodies in Australia have taken bold steps to incorporate planetary health and sustainability concepts within curricula to ensure graduates understand the connection between human health and the health of the environment and planet [10]. In doing so, these professions are setting standards for tertiary healthcare curricula and establishing a benchmark for other professions to follow. Australasian medical schools began by collaboratively developing and sharing planetary health‐related graduate outcomes and learning objectives [3]. The Australian Medical Council has subsequently included elements of planetary health within its National Framework and medical school standards [11]. The Australian Nursing and Midwifery Accreditation Council (ANMAC) is set to include planetary health within their accreditation standards [12], supported by a strong and cohesive body of work. For example, the Australian College of Nursing has created a four‐step stewardship approach, advocating strongly for nurses' role in planetary health through community consultation, rethinking service provision and developing the future workforce [13]. Within medicine and nursing, there is clear evidence of Australian health professions prioritising planetary health in practice, research, education and future strategy. The explicit inclusion of planetary health within standards is an important step as it requires institutions to take action to remain accredited. However, allied health professions in Australia are yet to instigate a cohesive approach, relying instead on innovative individuals to drive small changes, within their immediate contexts [14, 15, 16]. Despite isolated successes, the lack of higher level leadership and systematic endorsement has left planetary health on the fringes of allied health education.

Accreditation standards are powerful influences of curriculum reform and future workforce development [16] we have seen this with the integration of interprofessional education [17] and cultural safety [18]. The Aboriginal and Torres Strait Islander Health Curriculum Framework [2] was developed through extensive collaboration with stakeholders, offering a map to guide curriculum development and implementation in entry‐level health education, staged throughout programs from beginner, novice then entry‐level practice to enhance the cultural capabilities of health service delivery [19]. The successful implementation of the framework into curriculum is driven by accreditation requirements [3]. Apart from nutrition and dietetics, no other allied health disciplines have specific accreditation standards that relate to promoting aspects of planetary health and sustainability [20]. While it is critical to acknowledge inclusion of accreditation standards, it does not guarantee curriculum alignment or desired educational outcomes [19], standards provide shared benchmarks [21, 22]. The incorporation of planetary health cannot remain optional or even implied and must become an explicit accreditation requirement for allied health professions.

### 1.2 Adapting Global Recommendations to Local Contexts: The Role of Indigenous Knowledges

Successfully adapting planetary health recommendations to local contexts requires a conceptual shift toward thinking about the health of the environment in terms of stewardship principles: interconnection and inclusivity that go beyond Western beliefs and practices; advocacy and guardianship from within communities rather than left to individual champions; and conservation, promotion and generation through shared and mutual respect and responsibility [23, 24]. This may be facilitated by acknowledging the important role of Indigenous knowledges and perspectives in environmentally sustainable health care; perspectives that view land as inextricably connected to the giving and receiving of life [25] and are guided by Law of the Land [26].

Similar to the World Health Organisation's International Classification of Functioning, Disability and Health ICF [27], the determinants of health that are broadly adopted in the health sector focus on a Western‐centric view of health [26]. Broader views of health are helping to foreground social justice and health inequities in practice and education [6], although ecological determinants remain overlooked [25] Indigenous worldviews, grounded in principles of reciprocity and reverence for the Earth as a living entity [25] offer profound ecocentric insights. These perspectives are deeply intertwined with traditional knowledge systems, dynamic frameworks of know‐how, skills and practices that are cultivated, sustained and transmitted across generations [25]. Such systems not only inform ecological stewardship but also form an integral part of a community's cultural and spiritual identity and health [26].

### 1.3 A Way Forward

A focus on Western models and understandings of health and wellbeing creates curricula with a narrow focus, health professional graduates with limited capabilities for contemporary challenges, and arguably educators and higher education providers who have lost sight of community‐centred, Aboriginal approaches to a holistic understanding of health and wellbeing of communities and Country. Planetary health and sustainable healthcare practices cannot be separated from Aboriginal knowledges, which respectfully consider Country, communities and collectivism and instil understandings centring on the interconnectedness of all living beings on the planet [27]. Through Aboriginal health perspectives, we can develop culturally capable health graduates equipped to understand and action the changes needed for sustainable, community‐focussed planetary health practice.

Although many frameworks and statements exist to guide the planning, development and implementation of planetary health education for health professionals, very few consider Indigenous perspectives [28]. The Planetary Health Education Framework [29] and AMEE Consensus Statement [30] are notable exceptions. However, in a sea of guidelines and recommendations, neither of these have been widely adopted or implemented. In Australia, the medical and nursing accrediting and professional standards relating to planetary health do not explicitly mention First Nations perspectives, although it is claimed that ‘the Aboriginal and Torres Strait Islander and Māori knowledges of wellbeing and health care recognised in the standards can also contribute to the realisation of more sustainable and holistic practices’ [11] (p. 513).

This reflects an important, but we believe under‐appreciated, opportunity to use the Aboriginal and Torres Strait Islander Health Curriculum Framework [2] (herein referred to as The Framework) as a lens to explore planetary health and enact change in the educational formation of all future health professionals [2]. The Framework has played a central role in embedding culturally safe practices into Australian tertiary education curricula, underscoring how policy and strategic initiatives are instrumental in shaping and strengthening the health workforce. Using The Framework to explore and guide planetary health may help educators and accrediting bodies make planetary health an educational priority. The Framework's five capabilities of respect (cultural knowledge, humility and lifelong learning), communication (partnerships), safety and quality (population health), reflection and advocacy (leadership) align with planetary health capabilities and the transformative, inclusive and integrative approaches needed [31] (Table 1). Cultural safety involves a critical examination of the pervasive effects and inherent limitations of colonising mindsets across societal structures, fostering respectful engagement with multiple worldviews. Viewed through this lens, Aboriginal knowledges are recognised as vital in illuminating the deep interconnection between people, land and waters. As climate change continues to disproportionately affect Indigenous communities, initiatives such as The Framework play an essential role in advancing holistic, community‐centred responses [32]. Collectively, these approaches underscore the interconnectedness of human and environmental well‐being, promoting a more inclusive and culturally grounded vision of planetary health.

**TABLE 1:** Ways in which the Aboriginal and Torres Strait Islander Health Curriculum Framework (The Framework) [2] may help health professions to think about and integrate planetary health.

Element from the framework	Ways The Framework may provide a lens to view and integrate planetary health in Australian health professional curricula
Respect	Indigenous perspectives emphasise deep respect for Country, the land, water and all living beings, through ways of knowing, being and doing This worldview supports planetary health by recognising the Earth as a living entity deserving of care Health professionals learn to respect ecological knowledge systems, promoting sustainable living and environmental stewardship
Communication	Effective communication in Indigenous health involves listening deeply and sharing stories Storytelling can help weave scientific and cultural knowledge, making environmental health issues more relatable for diverse communities
Safety and quality	Quality care in Indigenous health includes culturally safe and holistic approaches, which can be expanded to include environmental determinants of health Planetary health encourages systems thinking, understanding how environmental degradation affects health outcomes Integrating this into quality standards ensures health care is both culturally and ecologically informed
Reflection	Reflection encourages practitioners to critically examine their values, biases and practices. It opens space to consider how colonial legacies have shaped both health and environmental degradation Reflective practice can lead to transformative learning, helping professionals reimagine their roles not just as caregivers, but as stewards of planetary wellbeing
Advocacy	Indigenous health frameworks promote advocacy for social and environmental justice. This aligns with planetary health's call to address climate change, pollution and biodiversity loss as health issues Health professionals can become advocates for sustainable policies, especially in vulnerable communities disproportionately affected by environmental harm

## 2 Conclusion

All Australian health professionals have a pivotal role as advocates for climate justice, leaders in sustainable healthcare and as environmentally responsible global citizens. By recognising Indigenous peoples' deep‐rooted knowledge of their environments and sustainable practices, we can gain valuable insights into addressing climate change and promoting planetary health. Applying an Indigenous lens supports the meaningful integration of a holistic perspective within health curricula and, ultimately, into clinical practice. The climate crisis will not wait. Meaningful, inclusive action must begin now.

## Funding

The authors have nothing to report.

## Conflicts of Interest

The authors declare no conflicts of interest.

## Data Availability

Data sharing not applicable to this article as no datasets were generated or analysed during the current study.
